# Addressing COVID-19 vaccine hesitancy in rural communities: A case study in engaging trusted messengers to pivot and plan

**DOI:** 10.3389/fpubh.2023.1059067

**Published:** 2023-02-09

**Authors:** Melhaney Reichelt, John Patrick Cullen, Sara Mayer-Fried, Holly Ann Russell, Nancy M. Bennett, Reza Yousefi-Nooraie

**Affiliations:** ^1^Department of Public Health Sciences, University of Rochester, Rochester, NY, United States; ^2^Clinical and Translational Science Institute, University of Rochester Medical Center, Rochester, NY, United States; ^3^Susan B. Anthony Center, University of Rochester, Rochester, NY, United States; ^4^Center for Community Health and Prevention, University of Rochester School of Medicine and Dentistry, Rochester, NY, United States; ^5^Department of Medicine, University of Rochester School of Medicine and Dentistry, Rochester, NY, United States; ^6^Department of Family Medicine, University of Rochester School of Medicine and Dentistry, Rochester, NY, United States

**Keywords:** COVID-19, communication, rural health, community-engagement, trusted messengers, vaccine confidence

## Abstract

The pandemic declaration of COVID-19 in 2020 presented unique challenges, lessons, and opportunities for public health practice in the United States. Despite clear evidence of COVID-19 vaccine effectiveness, vaccine uptake and vaccine confidence remained low in many regions. Vaccine holdouts, or those who are vaccine hesitant, have been an increasingly difficult population to reach. Several factors influence vaccine hesitancy and behavior in rural areas, including health care access challenges, misinformation, political loyalties, and concerns regarding the perceived lack of trustworthy evidence and knowledge of long-term effects. In March 2021, the Finger Lakes Rural Immunization Initiative (FLRII) engaged stakeholders to address vaccine hesitancy in a nine-county region of rural New York known as the Finger Lakes. Driven by data collected from community partners, physicians, and local health departments regarding their biggest barriers and greatest needs, the FLRII team created an interactive program for trusted messengers (TMs) including a stakeholder panel, called the Trusted Messenger Forum (TMF). The TMF met every 2 weeks from August 2021- August 2022 to engage local TMs and disseminate up-to-date knowledge in real time. During forum sessions, TMs shared detailed accounts of their experiences combating vaccine hesitancy in their communities and supported one another in their efforts through positive interaction and reaffirming conversations. Collaborations between community stakeholders can form a scaffolding to support a rapid response to a variety of public health problems and result in impactful change. For researchers implementing community-based research projects, modeling stakeholder panels after trusted messenger forums can be effective for diversifying the scope of the project and reacting to emergent problems in real-time.

## Introduction: The COVID-19 pandemic and vaccine confidence

Over the last 2 years the COVID-19 pandemic influenced many aspects of our lives. From stay-at-home orders and mask mandates in Spring 2020, to COVID-19 vaccine mandates and recommendations in workplaces and communities in Fall 2021, healthcare workers and community members have had to rapidly adapt to changing landscapes (1, 2). However, despite compelling evidence of the effectiveness of vaccination and governmental and state-wide supports to help citizens access COVID-19 vaccines, vaccination rates were not increasing as expected. Defined by the World Health Organization as a “delay in acceptance or refusal of vaccines despite availability of vaccination services,” vaccine hesitancy has been considered a leading threat to global health. The reasons behind vaccine hesitancy are complex; though key factors include complacency, inconvenience in accessing vaccines, and lack of confidence (3). Since vaccine confidence (the belief that vaccines are safe and effective) and the decision to get vaccinated are embedded in social and cultural contexts, social influence is a potentially effective mechanism to improve vaccination beliefs and behaviors (4, 5). In general, vaccine decisions, including those related to COVID-19 vaccines, are heavily influenced by individuals' healthcare providers (e.g., primary care doctor or nurse). However, Studies have shown that trustworthy and influential individuals are also an important agent of change to improve vaccination (6). Trusted messengers potentially belong to diverse social and professional groups, including healthcare providers, religious leaders, and trustworthy community members. Trusted messengers can be trained to use proven techniques that address vaccine hesitancy, including emphasizing personal benefit and pairing information about the collective benefit with concrete examples of how getting vaccinated protects vulnerable friends and family (7).

## Context: Rural people as vaccine hesitant

As COVID-19 vaccines became available in 2020, large and small-scale efforts were made to track vaccination uptake. Specifically, in upstate New York, data from the New York State Immunization Information System (2020–2021) was refined by the Rochester Regional Health Information Organization (RHIO) to determine regional COVID-19 vaccination rates and was analyzed to identify low vaccine uptake groups (e.g., under 70% eligible) (8). Data showed lower rates of COVID-19 vaccination in rural areas generally, and particularly among certain categories of individuals who were eligible for the vaccine. In response, the Centers for Disease Control and Prevention funded the University of Rochester Medical Center (URMC) Finger Lakes Rural Immunization Initiative (FLRII) initiative to address vaccine hesitancy in the rural communities of the Finger Lakes region in New York State (Genesee, Livingston, Monroe, Ontario, Orleans, Seneca, Wayne, Wyoming, and Yates Counties). The focus of FLRII was to leverage close partnerships with community partners, physicians, local health departments and other trusted messengers and collaborators to increase vaccine awareness, knowledge and acceptance of COVID-19 vaccine throughout the Finger Lakes.

## Methods: Trusted messenger-led response to vaccine hesitancy

### Identifying and responding to needs of trusted messengers

**Figure 1:**
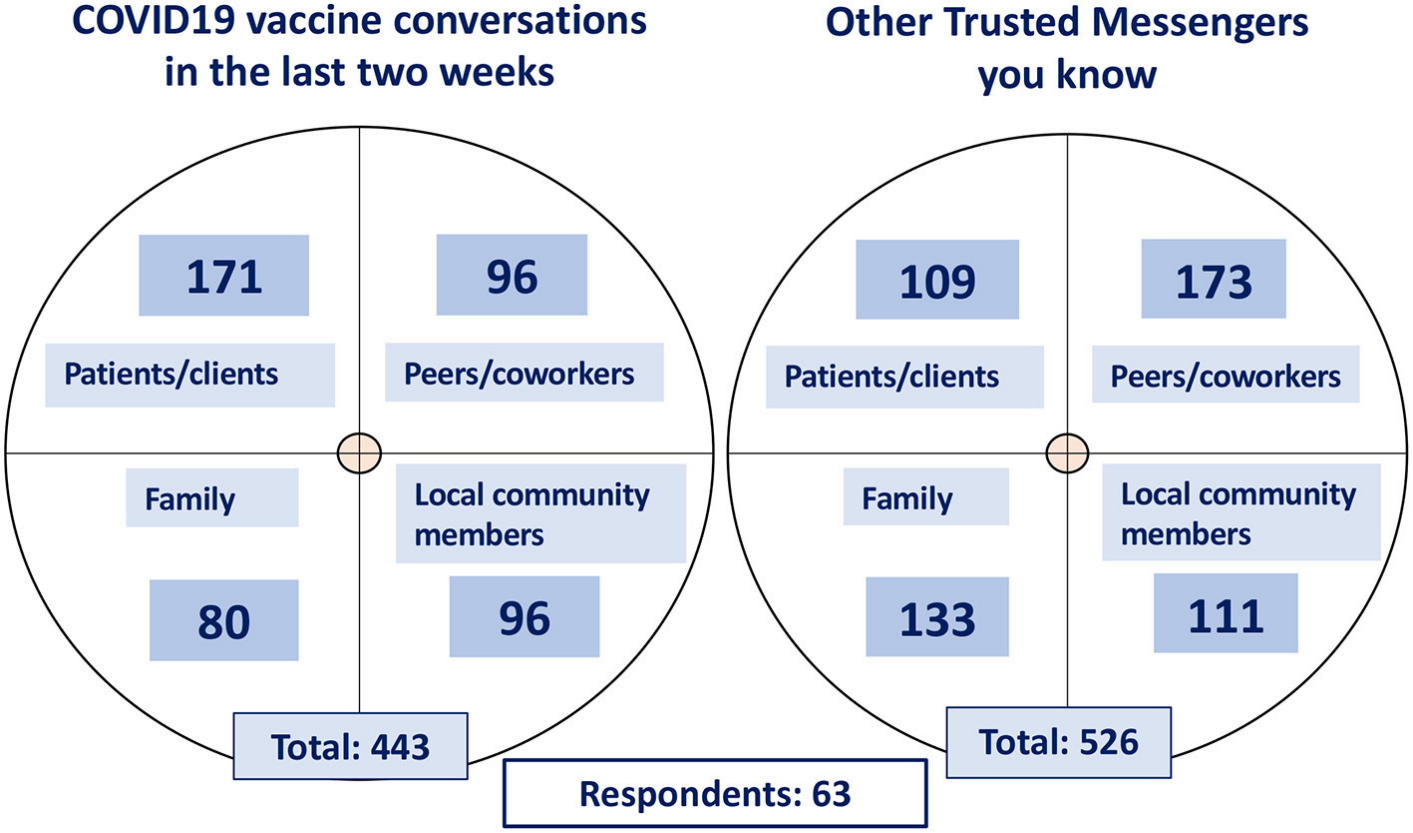
Trusted messenger communications and network connections.

To learn more about the influences, challenges, and interests of trusted messengers (TMs) the FLRII team developed and performed a mixed methods (qualitative and quantitative) need assessment and environmental scan to identify reachable influencers (TMs) and to support the development and implementation of a tailored support program. During the first few months after the initiation of FLRII, an online survey was sent to HCPs who self-identified as TMs. We found that, in total, the 63 respondents were involved in 443 vaccine communications (in prior 2 weeks) and knew 526 other TMs; thus, highlighting the reach and potential impact of peer-to-peer influence and communications (Figure 1). Likewise, self-identified TMs reported that they felt they could be influential in vaccine decisions of their patients/clients, peers/coworkers, family, and community members, however, they also felt they did not have the confidence nor sufficient channels for COVID-19 vaccine communication.

In response, the FLRII team created a TM support program comprised of **three** interventions: a Theater for Vaccine Hesitancy (TVH) workshop, a TM toolkit, and a bi-weekly live Zoom stakeholder forum. The TVH workshop was informed by multiple disciplines and was created to train TMs to have persuasive and respectful conversations with COVID-19 vaccine-hesitant patients and clients (9). Toolkit materials were designed for TMs to rapidly answer frequently asked questions and de-bunk common myths about COVID-19 vaccines. Finally, the goal of the stakeholder, or trusted messenger forum (TMF) was to build an engaging learning collaborative to leverage information from TMs and support their efforts promoting COVID-19 vaccination. The FLRII trusted messenger program is accessible through flrii.urmc.edu.

### Engaging trusted messengers through interactive forums

Self-identifying TMs were recruited to the forum through the needs assessment survey, participation in the TVH workshop, email invite from FLRII team, or peer invitation. Once enrolled, TMs received bi-monthly emails containing social media and FLRII website links, updated toolkit materials, previous TMF meeting notes, and invitations to upcoming sessions. By summer 2022 these emails were sent monthly. Trusted messenger forums were held over the course of 1 year, from August 2021 to August 2022, and were attended by health care providers, case managers, pharmacists, community based-organization staff, and community health workers. The frequency of sessions was continuously modified to match the needs of attendees: for the first 7 months TMFs were held bi-weekly and were reduced to once a month during the last 3 months of the FLRII grant. Sessions were held on Thursday evenings for 30 min and started with an introduction of FLRII members and TMs in attendance followed by TMs' open discussion and reflection. Session by session and total counts of TMF attendance are provided in Table 1.

**Table 1:** Trusted messenger forum attendance details.

**SESSION–SESSION**		
**Date**	**Participant (#)**	**Role (type)**
Aug 2021	3	MD, CCO
Oct 2021	3	RN, CCO
Oct 2021	2	RN
Nov 2021	2	RN
Dec 2021	6	RN, CM, CBO, NPO
Jan 2022	3	CM, CBO, NPO
Jan 2022	8	CM, CHW, CBO, NPO
Feb 2022	6	CM, CHW, CBO, NPO, Student
Feb 2022	1	CBO
Mar 2022	5	CM, CHW, CBO, NPO, PCN
Mar 2022	3	CM, CBO, NPO
Mar 2022	3	CM, CBO, NPO
Apr 2022	1	CM
May 2022	2	CM, RN
Jun 2022	1	CM
Jul 2022	-	-
Aug 2022	1	CM
**TOTALS (#)**		
**Session**	**Participant**	**Role (#/type)**
17	17	Two medical doctor (MD)
		Three nurse (RN) (PCN)
		One pharmacy (CCO)
		Two not for profit organization (NPO)
		One case manager (CM)
		One student
		Two community health worker (CHW)
		Five community based organization (CBO)

The meaning of the symbol # is to represent number.

Predominantly structured as listening sessions for the FLRII team, TMs attended forums to share their experiences, express frustrations, celebrate success stories, and build new and/or strengthen existing networks (10). In some cases, the TMF operated as a support group for TMs to reflect on the challenges of the pandemic and to confide in one another about how hard things had been. Rather than solely have the FLRII team lead, TMs served as mediators of conversation. This style encouraged organic conversation related to the different contexts that impacted their work: inequities, policies and practices, and attitudes/knowledge of hard-to-reach populations. The FLRII team was also interested in making COVID-19 information understandable and accessible for TMs and their communities.

Although the original intention was to record meetings, the FLRII team recognized it added a formality that didn't support unreserved communication. Instead, field notes were taken to capture the diverse experiences of TMs who attended. Two investigators (MR, SMF) took field notes and memos related to each TMF session. MR compiled the field notes and developed qualitative themes, following the thematic analysis approach (11). We categorized conversations from each session based on a thematic framework to capture the dynamics and products of sessions. Detailed accounts of session-by-session conversations in relation to barriers and proposed solutions are provided as Supplementary Tables 2, 3.

In this community case study report we will focus on presenting our experience with developing and coordinating the TMF and will provide recommendations for similar efforts seeking to increase and sustain the engagement of community stakeholders to inform implementation efforts.

## Results: Barriers and solutions to COVID-19 vaccination efforts

The following three cases will present a summary of specific barriers and solutions to COVID-19 vaccination efforts identified by trusted messengers during the forum sessions. The cases are broken into access and distributing vaccines, vaccine acceptance and knowledge, and vaccine communication.

### Accessing and distributing COVID-19 vaccines

#### Barriers

Low bandwidth in rural health centers made it hard for proper COVID-19 outreach measures, and the smaller, more rural health care practice facilities were initially unable to provide routine vaccination during office visits. Because of this, out-patient, or pop-up, clinics became the primary means for COVID-19 vaccination. TMs reported that pop-up vaccine clinics were not ideal: providers preferred to vaccinate during scheduled encounters (such as during office visits) and patients preferred to get vaccinated by someone with whom they had an existing relationship. Organizing a pop-up clinic was a burden for some community-based TMs and their attempts were often unsuccessful. Some TMs reported that there was a lack of qualified vaccinators in the area, and that those that were willing to volunteer required extensive training. Additional questions were raised regarding the best locations, recruitment of patients, and staffing.

#### Solutions

To address concerns about setting up successful pop-up vaccine clinics one TMF session focused solely on the best practices for organizing a clinic. During the session, one of the TMs delivered an informative lecture on the “easy” steps for planning up a clinic. TMs were advised to pick a location based on where their target population frequently gathers; to exhaust all options to spread the word about the event; and that while collaborations are a good thing, “less is more,” and TMs should refrain from making vaccination clinics into another event by overburdening the clients through educational activities. Advice was also provided on how to prepare staff for the clinic. This included meeting with volunteers to discuss where and when the event would be, who the provider would be, and individual assignments for volunteers. It was suggested that an event floorplan and daily itinerary be handed out to all clinic staff before the event. Additional key strategies for success included paying attention to details (such as checking CDC guidelines regularly) and making sure to have a backup plan (such as reschedule date or assign extra staff on site).

Most importantly, TMs discussed how to increase trust and initiate long-lasting relationships by demonstrating care for the overall health of a patient. This included follow-up calls in which vaccine discussions were placed second to the patient's reported priorities and urgent needs. TMs were advised to begin by first asking how the individual was doing and then offer to answer any questions. At the end, TMs should receive confirmation that their patient knows a follow up appointment is needed and if possible, ask if the patient knew of any family and friends in need of COVID-19 vaccines. The presentation was received very well by attending TMs, and all materials were shared following the session.

### COVID-19 *v*accine acceptance and knowledge

#### Barriers

TMs revealed that some community members lacked awareness of the realities of COVID-19's risks (severe symptoms, long-term effects, death). Whether this was due to an inability to access up-to-date reliable and trusted information, or an overload of conspiracy theories, many TMs had a difficult time efficiently and accurately addressing patient questions about COVID-19. For many TMs, mistrust and misinformation were believed to be the strongest barriers affecting vaccine acceptance and knowledge. Constantly changing health guidelines and competing messaging sources amplified feelings of hesitancy and doubts (disbelief in vaccine effectiveness) in already hard to reach or distrustful populations. As a result, TMs felt that there was no adequate way to respond to their patient's concerns. Many TMs reported problems finding accessible and up-to-date resources that would address their specific needs: such as vaccine dosing and isolation/quarantine guidelines, vaccination schedules and vaccination status criterion. Additionally, any resources that did exist were spread across multiple websites, took too long to find, and did not always have the information TMs were seeking.

#### Solutions

Informed by this conversation, FLRII was able to identify specific vaccine hesitant populations: people who have strong political views, women of reproductive age, individuals who are pregnant or nursing infants, parents of young children, and migrant farm workers. These findings were used by the FLRII team to develop materials for the interventional toolkit that could be used by TMs to succinctly address vaccine hesitancy among community members, patients, and colleagues. Toolkit materials included a quick response (QR) code and digital hyperlink frequently asked question (FAQ) document that could be easily accessible on the FLRII program website. Over the course of the year, TMs revealed new concerns and reminded the team of continued beliefs and attitudes of vaccine hesitant patients. This knowledge was applied to continuously modify the toolkit. The final toolkit includes material containing information related to vaccine ingredients, pregnancy and fertility, pediatric vaccines, multisystem inflammatory syndrome in children (MIS-C), and booster updates. Additionally, we created a Spanish language version to help us reach migrant communities and disseminate reliable information in their primary language.

### COVID-19 vaccine communication

#### Barriers

TM's reported that conveying the importance and urgency of COVID-19 vaccination was challenging. Even when vaccine providers had existing relationships with patients, TMs disclosed that initiating conversations about COVID-19 often felt difficult and impersonal, with no natural way to start. Additionally, TMs felt that not all health care workers were passionate about COVID-19 vaccines, and therefore avoided having the difficult conversations with vaccine-hesitant patients. TM's believed that not all individuals could be influenced by the same messages, and conversational approaches needed to be flexible enough to respond to the varied needs of vaccine hesitant individuals. This was a skill that many TMs felt they and/or their coworkers lacked. This increased tension between staff members, and many TMs reported work-place frustration and fatigue.

#### Solutions

The open forum became an opportunity to practice the sometimes-challenging interactions TMs faced around COVID-19 vaccines. TMs were encouraged to talk about examples of difficult conversations, or reasons for hesitancy they had encountered. In one session, messengers were invited to participate in a modified version of the TVH workshop and were given the opportunity to role play having conversations with vaccine hesitant individuals. The conversation topic (long-term effects, pediatric vaccines, booster shots, etc.) and reason for hesitancy (confusion, mis/distrust, etc.) was determined by the group based on the common misinformation and beliefs spreading at the time in the TMs' communities (9). TMs supported each other by providing feedback and advice on conversational cues. Ways to change the outcomes of sometimes challenging conversations included: being considerate, not using a dismissive or harsh tone, actively listening to patient concerns, and possibly sharing a personal vaccination story. Most importantly, TMs came to understand that it takes constant effort to get their message across and reported that it was necessary to be consistent and persistent. Communication between TMs navigating similar situations helped increase a sense of comradery among attendees, and partnerships among members extended outside the forum sessions. By talking through and clarifying moments of uncertainty, TMs were able to recognize defining moments for change and left the sessions feeling more confident in their ability to successfully do their work.

## Discussion

The forum was always intended to be a resource and not a burden. Though the forum sessions were an added activity at the end of a day, attendance was voluntary, and an honorarium was offered to attendees. Accordingly, TMs reported that the TMF was valuable: conversation was naturally engaging, and the sessions never felt overwhelming. Messengers expressed their willingness and desire to attend, and regularly returned to contribute to the discussion. Although not all those recruited attended the sessions, it is likely that this was at least in part due to the shift in staffing levels and the increased sense of burden, fatigue and frustration felt by health care workers and CBO staff. It is noteworthy that engaged TMs have social networks that extended far beyond the FLRII initiative (Figure 1), and the ideas/knowledge shared in the forum sessions have the potential to reach more TMs than just those who were recruited. Furthermore, the identities of these additional connections, as family members, patients, and community members, reinforces the idea that trusted messengers belong to diverse social and professional groups.

Importantly, the low-stakes forum was not only a fun and unique experience but was also a non-judgmental environment that spurred TM growth and confidence. Trusted messengers who engaged in both the TMF and the TVH workshop endorsed feeling more confident when discussing vaccines with their patients after engagement with the interventions. More specifically, TMs who participated in the TVH workshop and took the post-intervention survey reported an impact on patient decisions to get vaccinated: 45% of respondents judged that their patients were more likely to get vaccinated and 29% became vaccinated due to a change in conversational approach (9).

While we did not perform a formal evaluation of the TMF, we found that small, informal meetings with engaging dialogue have the power to provide meaning, elicit engagement, and inform interventions aimed at improving health outcomes. The forum also provided an opportunity for quick dissemination of resources and up-to-date information that could be used to address barriers in real-time. Finally, participation in TMFs resulted in the formation of multi-directional relationships that functioned to empower messengers and support the FLRII team in development and implementation of their public health intervention.

### Public health implications

Sustainable efforts are needed to establish transparent relationships between communities and health professionals to lay the foundation for future public health campaigns. Considering the increased need for effective strategies to implement health projects operating in challenging and rapidly changing landscapes, the trusted messenger forum can serve as an adaptable and critical tool for creating spaces for connection. For future public health professionals and researchers conducting quality improvement or community-engaged research projects, adoption of a stakeholder panel is suggested. Incorporating more voices and expanding the messenger network can help to foster trust, open channels of communication, and generate an up-to-date knowledge base between health research teams and residents living/working in communities. Additionally, stakeholder panel topics can be diversified and broadened to include health dialogue that goes beyond the COVID-19 virus. This could include conversations about other emerging viruses (such as monkeypox), reproductive justice rights, chronic disease prevention, gun violence, mental health and wellness, and many more pertinent topics.

Involving trusted messengers as co-developers can increase the applicability and feasibility of health projects. We were able to offer financial compensation for the time spent by TMs as this project was grant funded, however this is not necessarily a sustainable option. Institutions can consider the use of continued education credits or other forms of compensation to increase participation and willingness of community stakeholders and to fairly acknowledge the time spent on this work. As many academic institutions are moving to join forces with the community to promote health equity, it is important to employ the principles of community-based participatory research, a collaborative approach that involves community members during all phases of the research process/initiative development and recognizes the unique strengths that each member of the collaboration brings (12).

## Data availability statement

The original contributions presented in the study are included in the article/Supplementary material, further inquiries can be directed to the corresponding author.

## Ethics statement

The University of Rochester Research Subjects Review Board (RSRB) approved exemption status for the study (#00007287).

## Author contributions

MR and SM-F took comprehensive field notes during the session. MR performed thematic analysis of field notes and was the primary author of this manuscript. JC contributed with comments and paragraphs. SM-F provided comments and contributed 1–2 sentences. HR and NB provided verbal and written feedback. RY-N is the corresponding author and primary investigator for this manuscript. All authors were members of the FLRII project team, participated in the trusted messenger forum sessions, and contributed to writing this manuscript.
